# Supplementary material for: Assessment of ethanol and nicotine interactions using a reinforcer demand modeling with grouped and individual levels of analyses in a long-access self-administration model using male rats
Source: Front Behav Neurosci. 2023 Nov 30;17:1291128. doi: 10.3389/fnbeh.2023.1291128 (PMC10720750; doi:10.3389/fnbeh.2023.1291128)
Supplement: Supplementary file 1 [file Data_Sheet_1.pdf]

Supplemental Tables

Table S1. Ethanol demand parameters predict responding for ethanol on the PR schedule of reinforcement

	Dependent variable:					
	Total Active Lever Presses					
	(1)	(2)	(3)	(4)	(5)	(6)
EV	224.139**					-71.562
	p = 0.009					p = 0.861
Alpha		-116.450				-53.937
		p = 0.092				p = 0.473
Q0			18.305			10.615
			p = 0.108			p = 0.730
Omax				9.150**		
				p = 0.009		
Pmax					15.462**	16.081
					p = 0.010	p = 0.408
Constant	76.620*	181.030***	92.126	76.620*	47.018	44.410
	p = 0.045	p = 0.0001	p = 0.065	p = 0.045	p = 0.286	p = 0.675
Observations	14	14	14	14	14	14
R^2	0.448	0.219	0.201	0.448	0.439	0.532
Adjusted R^2	0.402	0.154	0.135	0.402	0.392	0.323
Residual Std. Error	87.981 (df = 12)	104.626 (df = 12)	105.785 (df = 12)	87.981 (df = 12)	88.672 (df = 12)	93.545 (df = 9)
F Statistic	9.722** (df = 1; 12)	3.360 (df = 1; 12)	3.025 (df = 1; 12)	9.722** (df = 1; 12)	9.385** (df = 1; 12)	2.554 (df = 4; 9)
Note:						*p<0.05; **p<0.01; ***p<0.001

Table S2. Nicotine demand parameters do not predict responding for ethanol on PR schedule of reinforcement

	Dependent variable:					
	Total Active Lever Presses					
	(1)	(2)	(3)	(4)	(5)	(6)
EV	52.178					23.330
	p = 0.575					p = 0.926
Alpha		160.164				1,249.787
		p = 0.915				p = 0.577
Q0			-0.251			5.340
			p = 0.979			p = 0.667
Omax				1.877		
				p = 0.575		
Pmax					0.968	1.650
					p = 0.470	p = 0.633
Constant	131.796*	150.533**	153.899**	131.796*	131.871**	70.519
	p = 0.017	p = 0.002	p = 0.004	p = 0.017	p = 0.008	p = 0.539
Observations	13	13	13	13	13	13
R^2	0.030	0.001	0.0001	0.030	0.049	0.103
Adjusted R^2	-0.059	-0.090	-0.091	-0.059	-0.038	-0.345
Residual Std. Error	102.521 (df = 11)	104.014 (df = 11)	104.068 (df = 11)	102.521 (df = 11)	101.511 (df = 11)	115.561 (df = 8)
F Statistic	0.335 (df = 1; 11)	0.012 (df = 1; 11)	0.001 (df = 1; 11)	0.335 (df = 1; 11)	0.562 (df = 1; 11)	0.230 (df = 4; 8)
Note:						*p<0.05; **p<0.01; ***p<0.001

Table S3. Nicotine demand parameters predict responding for nicotine on the PR schedule of reinforcement

	Dependent variable:					
	Total Nosepoke Entries					
	(1)	(2)	(3)	(4)	(5)	(6)
EV	112.180***					149.956
	p = 0.001					p = 0.139
Alpha		-1,116.411				-190.594
		p = 0.060				p = 0.673
Q0			-7.465*			-4.807
			p = 0.036			p = 0.075
Omax				4.034***		
				p = 0.001		
Pmax					1.392**	-0.909
					p = 0.003	p = 0.450
Constant	19.126	78.187***	80.672***	19.126	28.710*	43.273
	p = 0.105	p = 0.0003	p = 0.0002	p = 0.105	p = 0.022	p = 0.085
Observations	12	12	12	12	12	12
R^2	0.696	0.311	0.372	0.696	0.625	0.815
Adjusted R^2	0.665	0.242	0.310	0.665	0.587	0.709
Residual Std. Error	24.206 (df = 10)	36.434 (df = 10)	34.769 (df = 10)	24.206 (df = 10)	26.881 (df = 10)	22.569 (df = 7)
F Statistic	22.872*** (df = 1; 10)	4.510 (df = 1; 10)	5.932* (df = 1; 10)	22.872*** (df = 1; 10)	16.654** (df = 1; 10)	7.704* (df = 4; 7)
Note:						*p<0.05; **p<0.01; ***p<0.001

Table S4. Ethanol demand parameters do not predict responding for nicotine on the PR schedule of reinforcement

	Dependent variable:					
	Total Nosepoke Entries					
	(1)	(2)	(3)	(4)	(5)	(6)
EV	-4.678					-339.004
	p = 0.930					p = 0.180
Alpha		-12.065				2.481
		p = 0.754				p = 0.961
Q0			1.018			28.615
			p = 0.827			p = 0.198
Omax				-0.191		
				p = 0.930		
Pmax					2.231	5.972
					p = 0.633	p = 0.315
Constant	50.963*	52.339**	46.519*	50.963*	37.255	8.654
	p = 0.020	p = 0.007	p = 0.038	p = 0.020	p = 0.221	p = 0.852
Observations	12	12	12	12	12	12
R^2	0.001	0.010	0.005	0.001	0.024	0.263
Adjusted R^2	-0.099	-0.089	-0.094	-0.099	-0.074	-0.158
Residual Std. Error	45.032 (df = 10)	44.817 (df = 10)	44.936 (df = 10)	45.032 (df = 10)	44.514 (df = 10)	46.225 (df = 7)
F Statistic	0.008 (df = 1; 10)	0.104 (df = 1; 10)	0.051 (df = 1; 10)	0.008 (df = 1; 10)	0.243 (df = 1; 10)	0.625 (df = 4; 7)
Note:						*p<0.05; **p<0.01; ***p<0.001

Table S5. Nicotine demand parameters do not predict responding for ethanol on the PR schedule of reinforcement when nicotine is available on FR1

	Dependent variable:					
	Total Active Lever Presses					
	(1)	(2)	(3)	(4)	(5)	(6)
EV	187.411					-694.670
	p = 0.243					p = 0.240
Alpha		-2,469.658				-2,986.862
		p = 0.295				p = 0.296
Q0			-26.007			-20.487
			p = 0.054			p = 0.193
Omax				6.740		
				p = 0.243		
Pmax					3.123	8.862
					p = 0.126	p = 0.233
Constant	215.944*	329.111***	366.375***	215.944*	214.602**	458.746*
	p = 0.015	p = 0.0005	p = 0.0001	p = 0.015	p = 0.006	p = 0.017
Observations	11	11	11	11	11	11
R^2	0.148	0.121	0.355	0.148	0.241	0.562
Adjusted R^2	0.053	0.023	0.283	0.053	0.157	0.270
Residual Std. Error	151.409 (df = 9)	153.786 (df = 9)	131.719 (df = 9)	151.409 (df = 9)	142.891 (df = 9)	132.958 (df = 6)
F Statistic	1.563 (df = 1; 9)	1.239 (df = 1; 9)	4.957 (df = 1; 9)	1.563 (df = 1; 9)	2.860 (df = 1; 9)	1.924 (df = 4; 6)
Note:						*p<0.05; **p<0.01; ***p<0.001

Table S6. Nicotine demand parameters do not predict responding for ethanol on the PR schedule of reinforcement when nicotine is administered noncontingently

	Dependent variable:					
	Total Active Lever Presses					
	(1)	(2)	(3)	(4)	(5)	(6)
EV	190.353					-656.208
	p = 0.283					p = 0.434
Alpha		-1,238.463				-745.906
		p = 0.647				p = 0.851
Q0			-17.387			-6.868
			p = 0.282			p = 0.739
Omax				6.846		
				p = 0.283		
Pmax					3.198	10.629
					p = 0.157	p = 0.314
Constant	209.700*	302.535**	337.047**	209.700*	209.454*	323.833
	p = 0.031	p = 0.004	p = 0.002	p = 0.031	p = 0.014	p = 0.182
Observations	10	10	10	10	10	10
R^2	0.142	0.028	0.143	0.142	0.234	0.360
Adjusted R^2	0.035	-0.094	0.036	0.035	0.138	-0.151
Residual Std. Error	166.815 (df = 8)	177.629 (df = 8)	166.763 (df = 8)	166.815 (df = 8)	157.637 (df = 8)	182.227 (df = 5)
F Statistic	1.327 (df = 1; 8)	0.226 (df = 1; 8)	1.333 (df = 1; 8)	1.327 (df = 1; 8)	2.445 (df = 1; 8)	0.704 (df = 4; 5)
Note:						*p<0.05; **p<0.01; ***p<0.001

Table S7. Ethanol demand parameters do not predict responding for nicotine on the PR schedule of reinforcement when ethanol is available on FR1

	Dependent variable:					
	Total Nosepoke Entries					
	(1)	(2)	(3)	(4)	(5)	(6)
EV	-35.236					532.108
	p = 0.458					p = 0.323
Alpha		-66.326				-510.050
		p = 0.445				p = 0.116
Q0			-1.438			-44.273
			p = 0.741			p = 0.297
Omax				-1.438		
				p = 0.458		
Pmax					-4.374	-47.422
					p = 0.499	p = 0.183
Constant	46.886^{*}	39.649^{*}	41.259	46.886^{*}	60.005	345.824
	p = 0.039	p = 0.015	p = 0.088	p = 0.039	p = 0.144	p = 0.128
Observations	9	9	9	9	9	9
R^2	0.081	0.086	0.017	0.081	0.068	0.683
Adjusted R^2	-0.050	-0.045	-0.124	-0.050	-0.065	0.365
Residual Std. Error	32.926 (df = 7)	32.840 (df = 7)	34.061 (df = 7)	32.926 (df = 7)	33.163 (df = 7)	25.594 (df = 4)
F Statistic	0.617 (df = 1; 7)	0.657 (df = 1; 7)	0.118 (df = 1; 7)	0.617 (df = 1; 7)	0.509 (df = 1; 7)	2.152 (df = 4; 4)
Note:						*p<0.05; **p<0.01; ***p<0.001
